# Eukaryotic translation initiation factor 3 subunit B promotes head and neck cancer via CEBPB translation

**DOI:** 10.1186/s12935-022-02578-y

**Published:** 2022-04-22

**Authors:** Chengzhi Xu, Yupeng Shen, Yong Shi, Ming Zhang, Liang Zhou

**Affiliations:** 1 Department of Otolaryngology-Head and Neck Surgery, Eye Ear Nose and Throat Hospital, Fudan University, No. 83 Fenyang Road, Shanghai, 200031 China; 2 Department of Otolaryngology-Head and Neck Surgery, The First Hospital of Hebei Medical University, Shijiazhuang, 050000 China

**Keywords:** Head and neck cancer, Eukaryotic translation initiation factor 3 subunit (B EIF3B), Translation, CEBPB, IL6R

## Abstract

**Background:**

Head and neck squamous cell carcinoma (HNSCC) is the sixth most common cancer type worldwide. Deregulation of mRNA translation is a frequent feature of cancer. Eukaryotic translation initiation factor 3 subunit B (EIF3B) has been reported as an oncogene; however, its role in HNSCC has yet to be fully elucidated.

**Methods:**

In this study, the clinical significance of EIF3B expression was analyzed based on TCGA datasets. Then, EIF3B expression was knocked down and its role in HNSCC was revealed. To explore the molecular mechanisms of EIF3B, we applied RNA sequencing and proteomics and acquired deregulated pathways. RNA immunoprecipitation (RIP) sequencing was conducted to reveal the target mRNAs of EIF3B, and TCGA datasets were used to validate potential targets of EIF3B.

**Results:**

Elevated expression of EIF3B was observed in the HNSCC cancer samples. The expression of EIF3B was significantly correlated with the patient’s sex, age, HPV infection status, T stage, N stage, perineural invasion status and survival status. EIF3B serves as a marker of an unfavorable HNSCC prognosis. EIF3B-silenced Fadu and Cal27 cells exhibited reduced cell numbers, and EIF3B knockdown induced apoptosis in both cell lines. The EIF3B-silenced cells demonstrated decreased invasion and migration capabilities, and the EIF3B knockdown group mice showed significantly decreased tumor volumes. The results show that EIF3B promotes CEBPB translation and activates the MAPK pathway and revealed that IL6R and CCNG2 are targets of EIF3B-regulated CEBPB translation.

**Conclusion:**

In summary, the results indicated that EIF3B is a novel oncogene in HNSCC that promotes CEBPB translation and IL6R expression, and these findings provide a link between the molecular basis and pathogenesis of HNSCC.

**Graphical Abstract:**

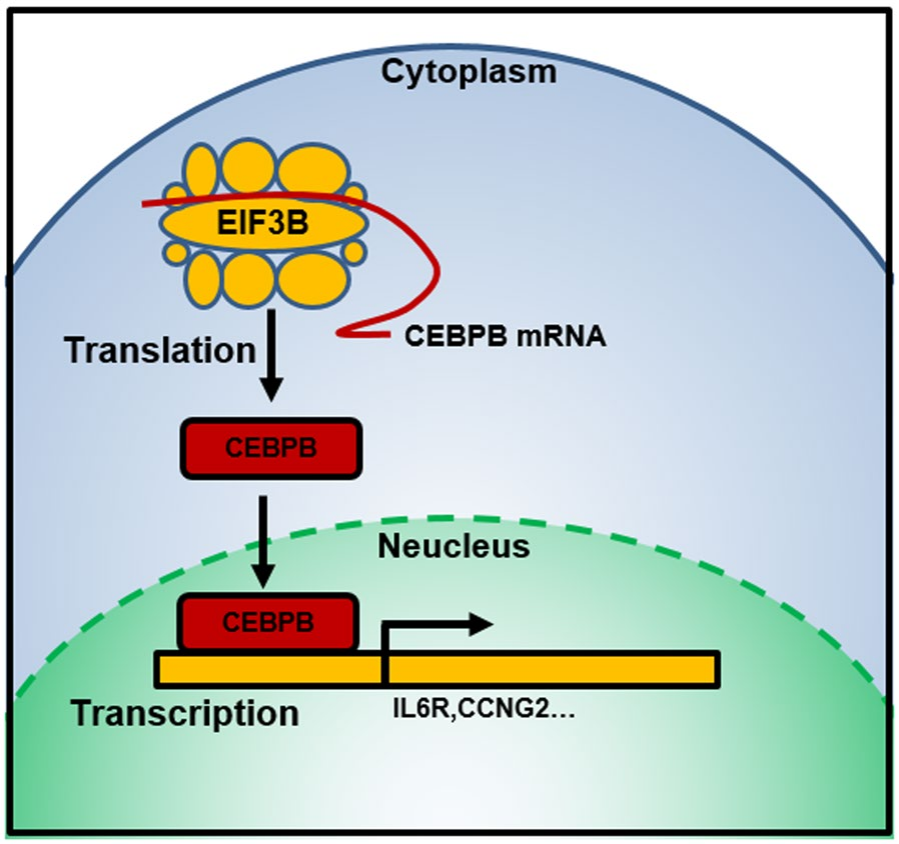

## Highlights

EIF3B is a prognostic biomarker for HNSCC risk;

EIF3B promotes HNSCC progression in vitro and in vivo;

EIF3B promotes CEBPB translation and activates the MAPK pathway;

IL6R and CCNG2 are targets of EIF3B-regulated CEBPB translation.

## Introduction

Head and neck squamous cell carcinoma (HNSCC) is the sixth most common cancer type worldwide [1], and its etiologic factors include smoking and excessive alcohol consumption; moreover, human papillomavirus (HPV) infection is a risk factor in some HNSCC types [2]. Tumor size, node involvement and smoking habits represent clinical prognostic factors [3]. Due to the limited surgical and adjuvant strategies, the 5-year survival of advanced HNSCC patients remains at < 50% [1]. Research on oncogenes in the pathogenesis and development of HNSCC would provide insights toward discovering novel therapeutic targets and improving the clinical outcome of patients.

Deregulation of oncogene and tumor suppressor mRNA translation is a frequent feature of cancer [4, 5], and translational regulation has been proposed as a potential therapeutic cancer target [6, 7]. EIF3B (eukaryotic translation initiation factor 3 subunit B) is an RNA-binding component of eukaryotic translation initiation factor 3 (eIF-3) complexes, which are required for several steps in the initiation of protein synthesis [8, 9]. The eIF-3 complex specifically targets and initiates the translation of a subset of mRNAs involved in cell proliferation, including cell cycling, differentiation and apoptosis [10]. The oncogenic role of EIF3B has been widely reported in gastric cancer [11], esophageal carcinoma [12] and bladder cancer [13]. However, the role of EIF3B in HNSCC remains unclear.

In this study, we aimed to reveal the role and underlying mechanism of EIF3B in HNCSS. The clinical significance of EIF3B in HNSCC was analyzed in TCGA and other public datasets. Then, the expression of EIF3B was knocked down, and its role in HNSCC was investigated in vivo and in vitro. To explore the molecular mechanisms underlying the effects of EIF3B, we applied RNA sequencing and proteomics analyses and identified deregulated pathways. Finally, RNA immunoprecipitation (RIP) sequencing was conducted to reveal the target mRNAs of EIF3B. This study reveals EIF3B as a novel oncogene in HNSCC that promotes transcription factor CEBPB translation and subsequent IL6R expression, and these findings provide a link between the molecular basis and pathogenesis of HNSCC.

## Materials and methods

### Data collection and procession

TCGA gene expression RNAseq (n = 546) and clinical phenotypes data of HNSCC (n = 612) was acquired from UCSC Xena website online tool (https://xena.ucsc.edu/). The clinical information was obtained through R. The exclusion criteria of clinical phenotypes data were as follows: (1) information on TNM was not provided or not applicable; (2) the clinical data were incomplete. All statistical analyses were performed using GraphPad v 8.0 software. The expression matrix was merged and normalized in R. Then we took the mean value of EIF3B expression and divide it into high and low expression groups according to this standard. Finally, we made statistics on the clinical information of samples with different EIF3B expression levels.

### Cell culture

Human HNSCC cell lines (Fadu and Cal27) were preserved in our lab. Fadu and Cal27 cells were cultured in Dulbecco's modified Eagle's medium (DMEM, Gibco, Gaithersburg, USA) supplemented with 10% fetal bovine serum (FBS; HyClone Laboratories, Logan, USA), 100 U/ml penicillin and 100 μg/ml streptomycin (complete media). All cell cultures were maintained as a monolayer culture at 37 °C in a humidified atmosphere containing 5% CO_2_.

### Lentivirus construction and infection

To knock down EIF3B expression in cell lines, a recombinant lentivirus expression vector (pGSIL-shEIF3B) containing a green fluorescent protein (GFP) tag was constructed. To generate lentivirus particles, the recombinant expression plasmid was cotransfected with a packaging plasmid system (psPAX2 and pMD2G) into Fadu and Cal27 cells, with viral particles collected after 48 h. Fadu and Cal27 cells were infected with the shEIF3B lentivirus vector or a negative control vector (Con) for 24 h. The shEIF3B target sequence was as follows:

shRNA1: 5′-GGAAGCAGATGGAATCGATTC-3′;

shRNA2: 5′-GGGAGAGAAATTCAAGCAAAT-3′;

shRNA3: 5′-GCAAATTCTTTGCCAGAATGA-3′.

### Quantitative real-time PCR

Total RNA was extracted from Fadu and Cal27 cells using TRIzol® RNA Isolation Reagent (Invitrogen, Carlsbad, CA) according to the manufacturer's instructions. Reverse transcription was performed using the PrimeScript™ RT reagent kit (Takara, Dalian, China). All mRNA levels were normalized to that of the housekeeping gene GAPDH. The following EIF3B primers were used in this study: 5′-GGACCCGACCGACTTGAGA-3′ (F) and 5′-TTGACCCGGAATGTGTGCTG-3′ (R). The sequences of the GAPDH primers were as follows: 5′-TGACTTCAACAGCGACACCCA-3′ (F), 5′-CACCCTGTTGCTGTAGCCAAA-3′ (R). All samples were treated under the same conditions and analyzed by qRT–PCR using SYBR Premix Ex Taq™ (Takara, Dalian, China) according to the manufacturer's protocol.

### Cell Counting Kit-8 (CCK-8)

Fadu and Cal27 cells were seeded into 96-well plates (2 × 10^4^ cells/well) and cultured for 12 h. After washing, the Fadu and Cal27 cells were incubated with 10% CCK-8 (Dojindo Molecular Technologies, Inc., Minato-ku, Tokyo, Japan), and the optical density was measured using a xMark Microporous Plate Absorption Spectrophotometer (Bio–Rad Laboratories, Inc., Hercules, CA, USA).

### Cell apoptosis assay

The apoptosis rate of Fadu and Cal27 cells was detected using an Annexin V 633 Apoptosis Detection Kit (Dojindo Molecular Technologies) following the kit instructions. The Fadu and Cal27 cells were seeded into 6-well plates (5 × 10^5^ cells/well) and cultured for 12 h, and then they were incubated with Annexin V, followed by propidium iodide (PI) buffer for 15 min at 25 °C in a dark room. Subsequently, apoptotic cells were quantified using a NovoCyte 1040 flow cytometer (ACEA Biosciences, Inc., Zhejiang, China).

### Transwell assay

Transwell chambers (Millipore Sigma, Burlington, MA, USA) and BD BioCoat Matrigel Invasion Chambers (Franklin Lake, NJ, USA) were used for cell migration and invasion assays. After incubation, the upper chamber residual cells were removed with cotton-tipped swabs, and then the cells that passed through the membrane were fixed with 4% paraformaldehyde and stained with 0.5% crystal violet. For each experiment, the number of migrating tumor cells was counted from 5 randomly selected fields. Each experiment was repeated at least 3 times.

### Wound healing assay

Migratory ability was assessed through a wound-healing assay. Fadu and Cal27 cells (2 × 10^5^) were seeded into a 6-well plate and allowed to reach confluence. Then, uniform wounds were scraped across the cell monolayer using a 200 µl pipette tip. Cells were rinsed with phosphate-buffered saline and cultured in the medium, and wound closures were observed after 24 h. The initial gap length (0 h) and residual gap length (24 h) after wounding were calculated from photomicrographs using an Olympus fluorescence microscope (Olympus).

### Nude mouse tumor xenograft assay

Female 4-week-old nude mice were obtained from the Slaccas Company. A total of 2 × 10^6^ cells from each group was injected subcutaneously into the mice 12 h after transfection (n = 5). For the same cell line, the normal control was implanted into the left posterior flank and the knockdown treatment was implanted into the right flank of each mouse. The tumor size was measured every week with calipers. Three weeks later, the mice were euthanized with 100 µL of a 10:1 mixture of ketamine (100 mg/mL) and xylazine (100 mg/mL) by injection into the lateral tail vein, and death occurred within approximately 1 min [14].

### Transcriptomic RNA sequencing and analysis

Total RNA was extracted as described above using the NucleoSpin RNA kit (Macherey Nagel, cat. no. 740955). RNA integrity was analyzed using a Bioanalyzer (Agilent, RIN: 9.7–10). Approximately 500 pg of RNA per sample was reverse-transcribed and amplified using a modified SMARTseq2 protocol (Rambow et al. 2018). Prior to generating sequencing libraries using the NexteraXT kit (Illumina, cat. no. FC-131–10), cDNA profiles were monitored using a Bioanalyzer. Sequencing was performed on the Nextseq500 platform (Illumina, SE75bp). Reads were then mapped to the human genome (hg19) using STAR (2.4.1b) and quantified with Subread (1.4.6-p2). The results were uploaded to the NCBI under the bioproject accession number PRJNA664253.

Differential analyses between EIF3B-silenced and WT samples were executed using the DeSeq2 pipeline. Genes with RNA expression fold change >  = 2 or < 0.5 and p values < 0.05 were defined as significantly differentially expressed proteins.

### Quantitative proteomics analysis

Proteomic methods were applied as described in previous publications. Briefly, proteins in the EIF3B-silenced Fadu cells and control cells were extracted and subjected to trypsin digestion. Then, a spectral library was generated and used for identification and quantitation. Data extraction was performed using Spectronaut X, and the ideal extraction window was dynamically determined based on the iRT calibration and gradient stability. The FDR cutoff at the precursor and protein levels was < 1%. Decoy generation was set to apply a random number of amino acid position swamps (min = 2, max = length/2). Otherwise, all the selected fragment ions passing the filters were used for quantification [15]. Proteins with expression fold change >  = 2 or < 0.5 and a p value < 0.05 were defined as significantly differentially expressed proteins. The mass spectrometry proteomics data have been deposited to the ProteomeXchange Consortium (http://proteomecentral.proteomexchange.org) via the iProX partner repository [15] with the dataset identifier PXD021532.

### RNA immunoprecipitation (RIP) sequencing

RIP sequencing was performed as previously described with minor modifications [16]. Briefly, Fadu cells were washed and harvested in ice-cold PBS and lysed in RIP lysis buffer. The lysates were incubated on ice for 10 min and centrifuged at 14,000 rpm for 15 min to clear cell debris. The supernatants were supplemented with NT2 buffer, EDTA to 15 mM, DTT to 1 mM, RNaseOUT and VRC and then immunoprecipitated with anti-EIF3B/Flag antibody at 4 °C overnight. The samples were washed five times with NT2 buffer supplemented with 15 mM EDTA. RNA was eluted with TRIsure (Bioline) according to the manufacturer's instructions. The isolated RNA from RIP was analyzed by RNA sequencing.

### Statistical analysis

All statistical analyses were performed using GraphPad v 8.0 software. Each experiment was performed in triplicate, and the data are shown as the mean ± SD, unless otherwise stated. The Kaplan–Meier method was applied as the survival analysis, and the log-rank test was used to estimate the differences in survival. P values < 0.05 were considered statistically significant.

## Results

### EIF3B acts as an unfavorable prognostic marker in HNSCC

**Fig. 1:**
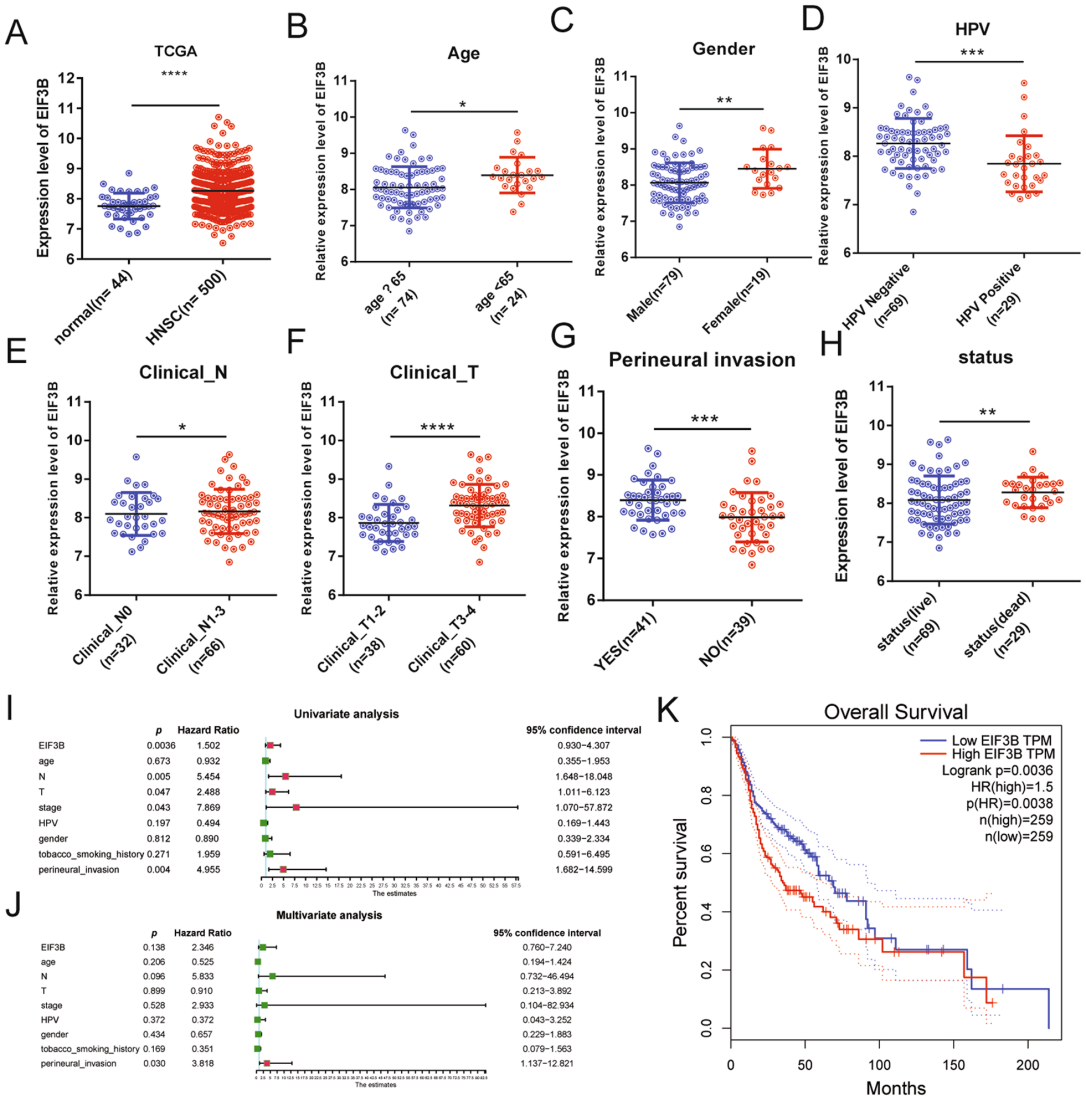
EIF3B is a prognosticator of HNSCC risk. In the TCGA HNSCC dataset, EIF3B shows significantly higher expression in cancer samples. EIF3B expression is significantly correlated with the patient’s clinical age (**B**), sex (**C**), HPV infection status (**D**), clinical T stage (**E**), clinical N stage (F), perineural invasion status (**G**) and outcome status (**H**). *, P < 0.05, **, P < 0.01, ***, P < 0.001, ****, P < 0.0001. **I** Univariate Cox analysis of EIF3B and other clinical parameters with HNSCC survival. **J** Multivariate Cox analysis of EIF3B and other clinical parameters with HNSCC survival. **K** Kaplan–Meier plot showing EIF3B as a prognosticator of HNSCC risk

To investigate the expression of EIF3B in HNSCC, we first checked its expression in the TCGA HNSCC dataset. As shown in Fig. 1A, EIF3B expression was elevated in the HNSCC cancer samples compared with the control normal samples. Then, we analyzed the relations between EIF3B expression and pathological parameters in HNSCC. As shown in Table 1 and Fig. 1B–H, EIF3B expression was significantly correlated with the patient’s sex, age, HPV infection status, T stage, N stage, perineural invasion status and survival status. Finally, the prognostic significance of EIF3B expression was analyzed with univariate and multivariate Cox regression analyses (Fig. 1J) and shown using a Kaplan–Meier plot (Fig. 1K). Patients with higher EIF3B expression showed significantly lower survival rates, suggesting that EIF3B serves as an unfavorable prognostic marker in HNSCC.

**Table 1:** Correlation of EIF3B expression with clinical parameters in HNSCC

Characteristics	EIF3B			
	Low (No. cases)	High (No. cases)	X^2^	p value
*Age*			7.946	**0.005**
≥ 65	6	18		
< 65	43	31		
*Gender*			5.288	**0.021**
Male	44	35		
Female	5	14		
*Stage*			3.199	0.074
Stage 1–2	13	6		
Stage 3–4	36	43		
*N*			0.742	0.389
N0	18	14		
N1–3	31	35		
*T*			17.193	**0**
T1–2	29	9		
T3–4	20	40		
*HPV*			14.154	**0**
Negative	26	43		
Positive	23	6		
*Tobacco_smoking_history*			0.763	0.858
Lifelong Non-smoker	11	8		
Current smoker	17	19		
Current reformed smoker for > 15 years	6	7		
Current reformed smoker for ≤ 15 years	13	15		
*Perineural_invasion*			7.167	**0.007**
NO	23	16		
YES	12	29		
*Status*			3.967	**0.046**
Live	39	30		
Dead	10	19		

Bold indicates p ≤ 0.05

### EIF3B promotes HNSCC proliferation and progression in vitro and in vivo

**Fig. 2:**
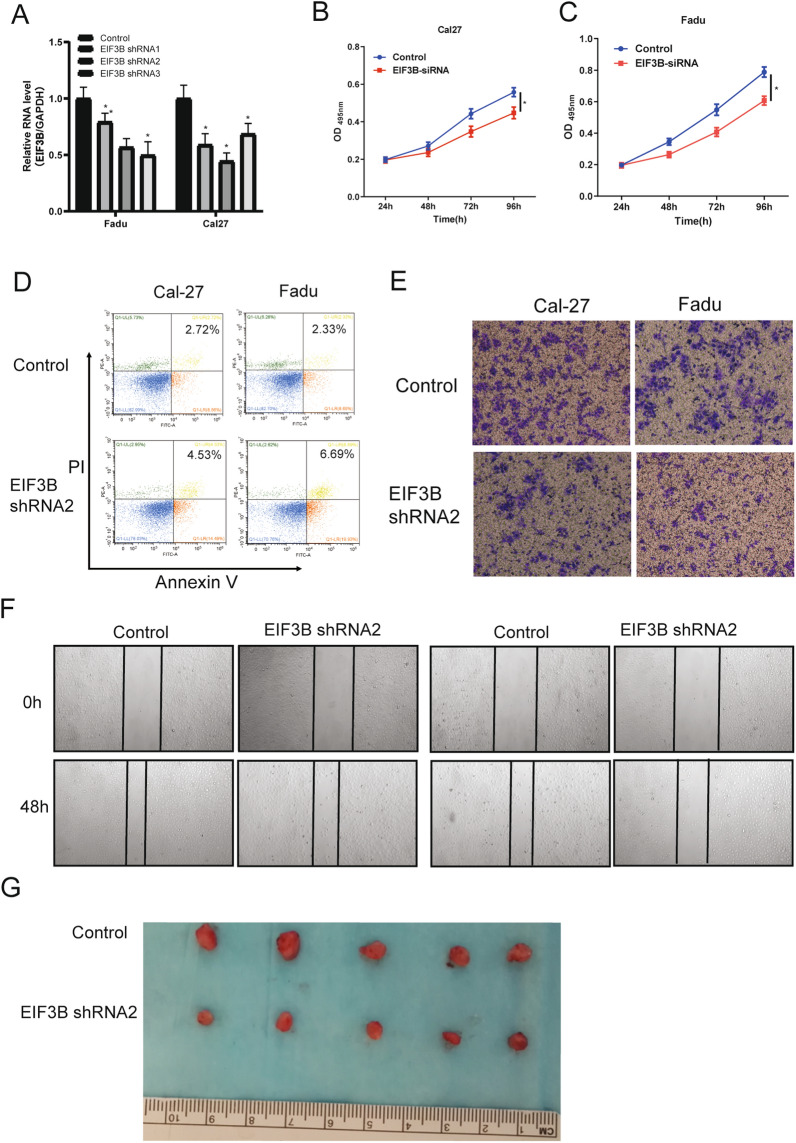
EIF3B knockdown reduces HNSCC in vitro and in vivo. **A** EIF3B was knocked down using shRNAs, and the knockdown efficiency was examined via qRT–PCR in Fadu and Cal27 cells. **B**, **C** CCK8 assay indicating that EIF3B knockdown attenuated the proliferation of Fadu and Cal27 cells. **D** Cell apoptosis rate increased in Fadu and Cal27 cells in the EIF3B knockdown groups. **E** Cell invasion examined with a transwell assay in Fadu and Cal27 cells. **F** Cell migration examined with wound healing assays in Fadu and Cal27 cells. **G** In vivo nude mouse xenograft results showed that EIF3B knockdown reduced the tumor volume of Fadu cells (n = 5). All assays were replicated three times. *, P < 0.05; **, P < 0.01

To explore the functional role of EIF3B in HNSCC, we first applied lentivirus-mediated EIF3B knockdown (Fig. 2A) in the Fadu and Cal27 cell lines. As shRNA2 shows the overall best silencing efficiency, it was chosen for subsequent phenotypic studies. As shown in Fig. 2B, C, EIF3B-silenced Fadu and Cal27 cells exhibited reduced cell numbers compared with the control cells, suggesting that EIF3B may contribute to cell growth in HNSCC. Then, the apoptosis rate in EIF3B knockdown and control cells was analyzed. As shown in Fig. 2D, the Annexin V/PI staining assay indicated that EIF3B knockdown induced apoptosis in both cell lines, especially in Fadu cells. Next, transwell and wound healing assays were conducted to explore the impact of EIF3B silencing on cell invasion and migration. The results showed that the EIF3B-silenced cells in both lines demonstrated a decreased invasion capability, as shown in Fig. 2E. Similar to the transwell assay results, EIF3B knockdown in the HNSCC cell lines led to greater differences in cell migration relative to the controls, implying that EIF3B knockdown also inhibited cell migration (Fig. 2F). As Fadu cells showed more pronounced overall tumor behavior than Cal27 cells, we chose Fadu cells for subsequent in vivo and mechanistic studies. Finally, we applied a nude mouse xenograft experiment using Fadu cells to explore the function of EIF3B in vivo. As shown in Fig. 2G, the tumor volume in the EIF3B knockdown group was significantly decreased, thus supporting the oncogenic role of EIF3B in HNSCC.

### Integrated analysis reveals CEBPB as a target of EIF3B translation

**Fig. 3:**
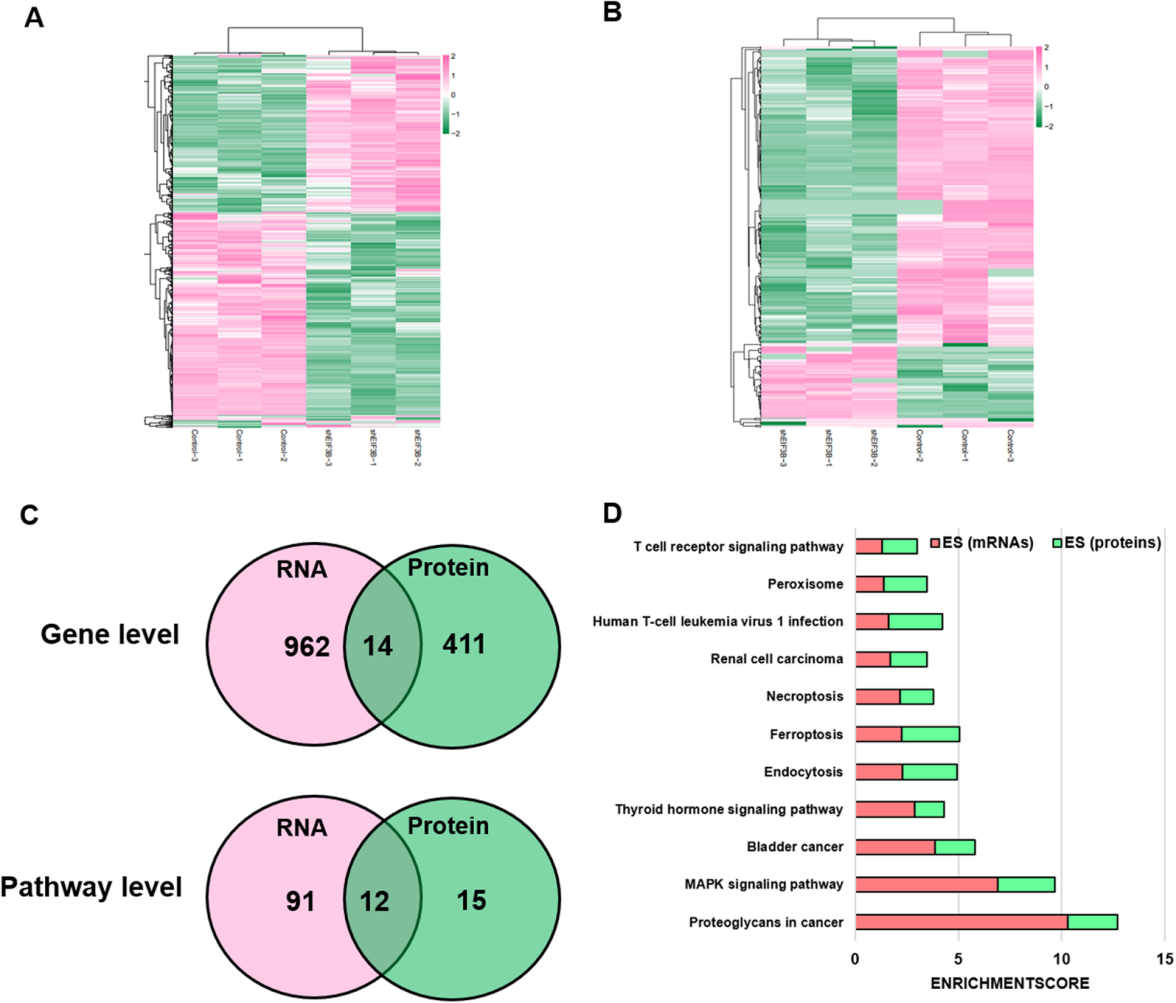
Integrated transcriptomic and proteome analysis reveals potential pathways of EIF3B. **A** Total of 976 mRNAs were significantly deregulated after EIF3B knockdown, and their expression is shown using a heatmap. **B** Total of 425 proteins were significantly deregulated after EIF3B knockdown, and their expression is shown using a heatmap. **C** At the gene level, 14 genes showed both deregulation at the mRNA and protein levels, and 12 common pathways were significantly enriched. **D** Twelve common enriched pathways are shown based on the pathway enrichment score (ES = -log10 P value)

**Fig. 4:**
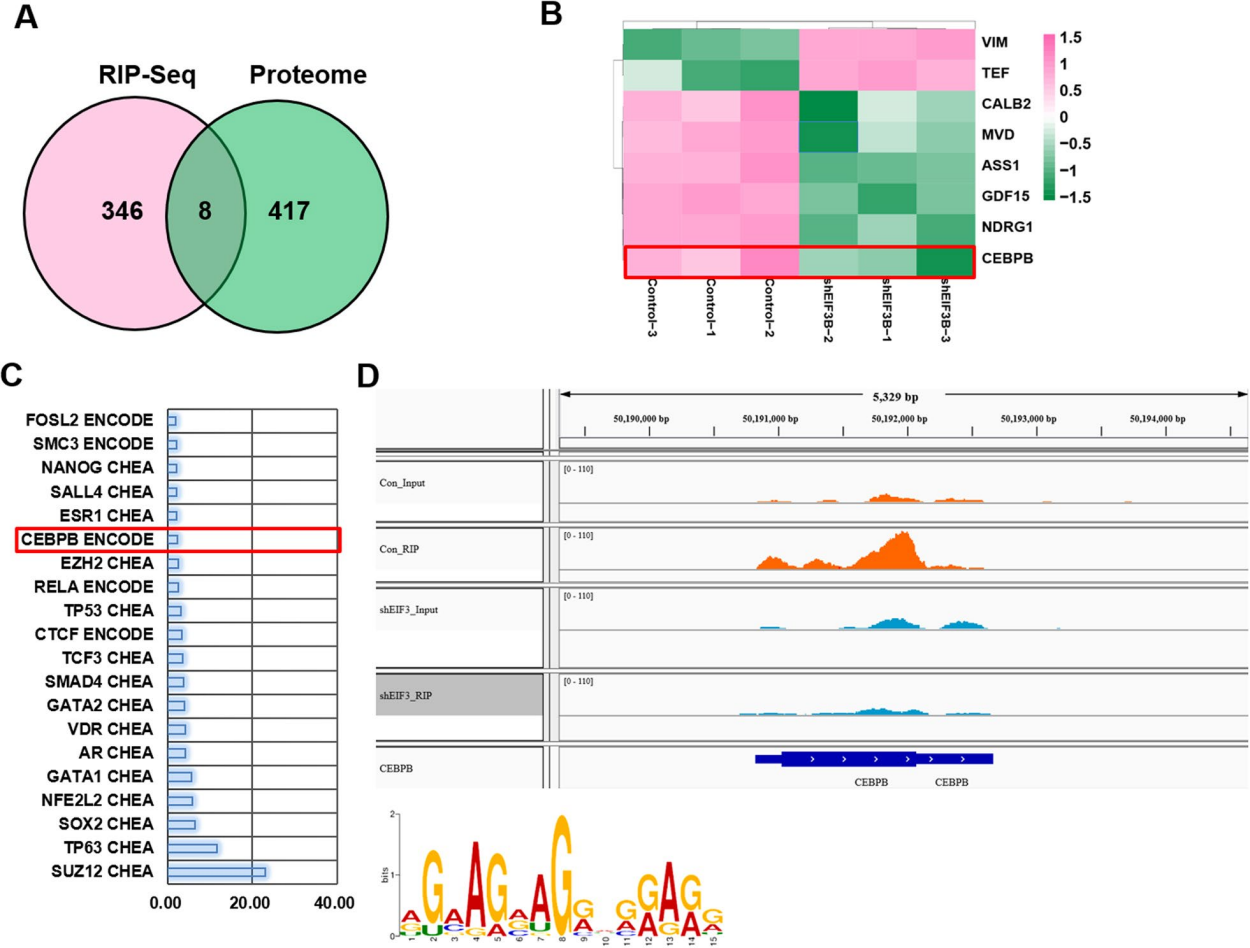
RIP-Seq analysis reveals CEBPB as a direct target of EIF3B. **A** RIP-Seq and proteome analyses reveal 8 genes that showed significant differential expression at the protein level (**B**) and direct mRNA binding affinity by EIF3B. **C** Significantly deregulated mRNAs were used for ENCODE and ChEA consensus transcription factor prediction, and the top 20 transcription factors are shown. Based on the RIP-Seq, proteome and RNA-seq results, the transcription factor CEBPB was proposed as a direct target of EIF3B. The binding motif and peaks of EIF3B with CEBPB mRNA are shown in **D**

To explore the molecular mechanism of the oncogenic role in HNSCC, we first applied RNA sequencing and label-free proteomics to EIF3B knockdown and control Fadu cells. RNA sequencing screened 976 significantly deregulated mRNAs (fold change >  = 2 or <  = − 2, P value < 0.05), as shown in Fig. 3A. Meanwhile, quantitative proteomics identified 425 significantly deregulated proteins among all 5195 proteins (fold change >  = 1.5 or <  = − 1.5, P value < 0.05), as shown in Fig. 3B. Then, the genes and significant pathways of the deregulated mRNAs and proteins were compared. As shown in Fig. 3C, only 14 genes (LAMB3, MYL9, NDRG1, FOSL1, ADAM8, SPRY4, HMOX1, RAB31, PIR, TGFB1, TEF, FAT2, ABR and GRB7) showed deregulation at both the mRNA and protein levels. At the pathway level, 103 and 27 significant (P < 0.05) pathways were identified for mRNAs and proteins, respectively. For mRNAs, the top 3 most significant pathways were associated with the pathways, proteoglycans, and transcriptional misregulation involved in cancer. For proteins, the top 3 most significant pathways were ribosome, RNA transport and ferroptosis. As shown in Fig. 4C, 12 common significant pathways were enriched at both levels. Based on the enrichment score (log10 P value), the 12 significant pathways at both levels are shown in Fig. 3D. In addition to the MAPK pathways, which are well-recognized pathways in cell proliferation and apoptosis, other cell death pathways, such as ferroptosis and necrosis, were significantly enriched, which explains the function of EIF3B in regulating cell proliferation and cell death.

EIF3B is a translation initiation factor that is involved in gene translation by binding to mRNAs. Since EIF3B plays an oncogenic role in HNSCC, the abnormal expression of EIF3B may influence target gene translation and protein expression. Therefore, we applied RNA immunoprecipitation and RNA sequencing (RIP-Seq) to identify potential EIF3B-binding mRNAs, and a total of 354 mRNAs were identified. Then, we compared the 354 mRNAs with 425 significantly deregulated proteins in the EIF3B silencing group and identified 8 common genes, namely, VIM, TEF, CALB2, MVD, ASS1, GDF15, NDRG1 and CEBPB (Fig. 4A), and their expression results are shown in Fig. 4B. Then, we examined studies on the oncogenic and tumor-suppressing roles of the 8 genes in HNSCC and finally chose CEBPB as a target candidate. CEBPB has been reported to confer radiation resistance in nasopharyngeal carcinoma [17]. As a transcription factor, CEBPB was reported to be translationally regulated by EIF6 [18] and regulate the MAPK pathway in cancer [19]. To validate the role of CEBPB at the mRNA level, we applied ENCODE and ChEA Consensus Transcription factor prediction and found that CEBPB was one of the most significant proteins (Fig. 4C). Finally, the binding peaks of EIF3B with CEBPB mRNAs and conserved motifs are shown in Fig. 4D. After EIF3B was silenced, the binding peaks were reduced compared with those of the control group. In summary, we identified CEBPB as a translational target of EIF3B through which EIF3B regulates the MAPK and other pathways in HNSCC.

### Transcriptional targets of EIF3B regulated CEBPB translation

**Fig. 5:**
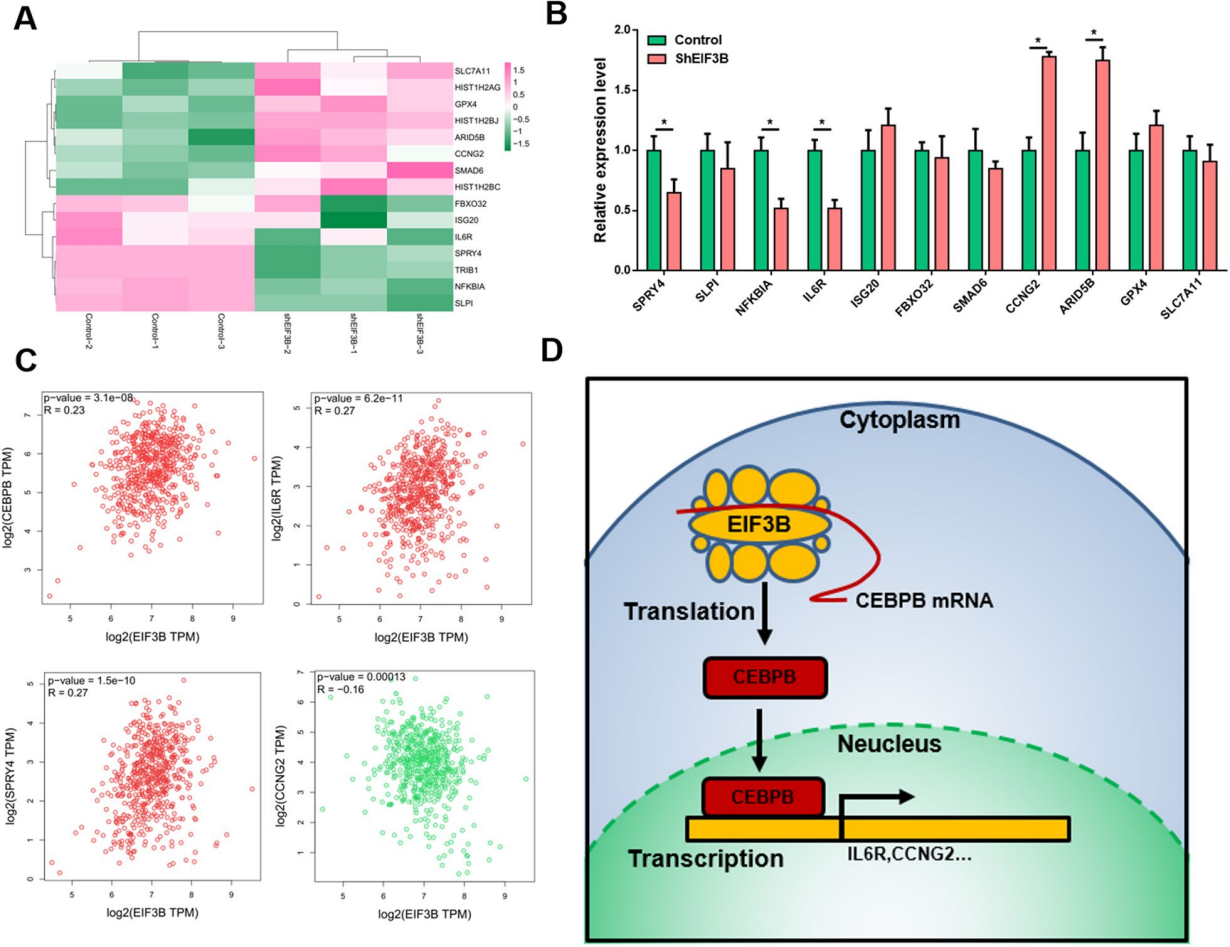
EIF3B regulates CEBPB target expression and its role in HNSCC. **A** Expression of the CEBPB targets in the control and EIF3B knockdown groups (shEIF3B) is shown using a heatmap. **B** qRT–PCR was applied to validate the expression of CEBPB targets, and SPRY4, NFKBIA, IL6R and CCNG2 showed a consistent trend with the RNA-seq results. **C** Coexpression of EIF3B and CEBPB targets was validated with the TCGA HNSCC dataset, and EIF3B showed a significant positive correlation with CEBPB, SLPI, IL6R, and SPRY4 and a negative correlation with CCNG2. **D** EIF3B promotes HNSCC progression by directly binding and promoting the translation of CEBPB and activating the transcription and expression of downstream targets (e.g., IL6R and CCNG2)

Finally, we explored the potential targets of EIF3B-regulated CEBPB translation. Fifteen genes were identified by ENCODE and ChEA Consensus Transcription factor prediction, and their expression in the EIF3B knockdown and control groups is shown in Fig. 5A. Then, a qRT–PCR assay was performed to validate 11 genes in EIF3B knockdown and control Fadu cells. As shown in Fig. 5B, 5 genes (SPRY4, NFKBIA, IL6R, CCNG2 and ARID5B) exhibited consistent significant differential expression. Finally, we calculated the coexpression coefficient of EIF3B with CEBPB and these 5 genes. As shown in Fig. 5C, EIF3B showed a significant positive correlation with CEBPB (R = 0.23, P = 3.1e−08), IL6R (R = 0.27, P = 6.2e−11) and SPYR4 (R = 0.27, P = 1.5e−10) but a significant negative correlation with CCNG2 (R = − 0.16, P = 0.00013). Finally, we proposed that the molecular mechanism of EIF3B in HNSCC involves promoting CEBPB translation and regulating targets, such as IL6R and CCNG2 (Fig. 5D).

## Discussion

Deregulated translation is characterized as an abnormal molecular cancer event, and related genes are emerging as potential therapeutic targets [6]. Studies are increasingly focusing on the family members of eukaryotic translation initiation factors in HNSCC progression and prognosis. In this study, we analyzed the clinical significance of EIF3B in HNSCC. Consistent with the other cancer types mentioned above, EIF3B showed significantly higher expression in HNSCC and patients with elevated expression showed worse prognosis, indicating that EIF3B represents a risk factor in HNSCC prognosis.

EIF4E is one of the most reported genes and has been reported to cooperate with c-Myc and promote HNSCC progression [20]. In mammals, the eukaryotic translation initiation factor 3 (eIF-3) complex comprises 13 subunits: EIF3A-M. The role of EIF3B as a biomarker of poor prognosis has been reported in gastric cancer [21], lung cancer [21, 22], glioma [23], ovarian cancer [24] and renal cancer [25]. EIF3B is also an oncogene in gastric cancer [11], lung cancer [21, 22, 26], osteosarcoma [27], esophageal squamous cell carcinoma [12, 28], leukemia [29] and renal cancer [25]. In HNSCC, the eIF-3 complex is recruited by DDX3 and promotes HNSCC metastasis by translating upstream open reading frames (uORFs) that contain oncogenic mRNAs [30]. We found that EIF3B-silenced Fadu and Cal27 cells exhibited reduced cell numbers. EIF3B knockdown induced apoptosis in both cell lines, and EIF3B-silencing led to decreased invasion and migration. Moreover, the tumor volume in mice of the EIF3B knockdown group was significantly decreased.

Interestingly, we also found that HPV-positive HNSCC patients showed significantly lower EIF3B expression. Major molecular differences are observed between HPV-positive and HPV-negative oropharyngeal cancers, and they underlie the major clinical differences between these cancer types [2]. However, whether HPV infection influences EIF3B expression remains to be explored. Another finding is the significant correlation of EIF3B expression with perineural invasion in HNSCC. Perineural invasion (PNI) is a mechanism of tumor dissemination that occurs via nerves, and it is associated with poor clinical outcomes [31]. To date, PNI markers, such as the neurotropic factors NGF and BDNF, have been proposed [32]. The significance of the EIF family member eIF-4E with perineural invasion was reported in colorectal cancer [33], and the correlation between EIF3B and PNI in HNSCC was first reported in this study. The significant coexpression of EIF3B with PNI markers also supported the conclusion of EIF3B as a PNI marker. In the future, we will explore the role of EIF3B in HNSCC perineural invasion.

CEBPB is a transcription factor widely reported in cancer. In nasopharyngeal carcinoma and HNSCC, CEBPB is involved in PGC1α-mediated radiation resistance [17, 34]. The abnormal translation of CEBPB [35] has been widely reported in macrophage migration [36], metabolic diseases [37] and leukemia [38]. EIF6 was previously described as a CEBPB translational regulator, mainly in adipogenic regulation [18]. We identified CEBPB as a translation target of EIF3B and proposed that its oncogenic role involved modulating downstream targets, such as IL6R and CCNG2.

Certain limitations that affected this work should be detailed. First, this study silenced the expression of EIF3B in the Fadu and Cal27 cell lines and assessed the effect of EIF3B overexpression. Second, this study proposed that EIF3B promotes HNSCC via CEBPB translation; however, a rescue assay should be performed to determine whether CEBPB protein upregulation would phenocopy the function of EIF3B and provide more solid evidence for the above conclusion. Finally, the clinical significance of EIF3B in HNSCC was mainly based on the public TCGA database. If possible, the significance may be validated with more HNSCC specimens and clinical data.

## Conclusion

EIF3B serves as a prognostic marker of hazard in HNSCC. Lentivirus-mediated EIF3B knockdown inhibited cell proliferation, migration, and invasion in vivo and in vitro. EIF3B promotes CEBPB translation and activates the MAPK pathway, and IL6R and CCNG2 are targets of EIF3B-regulated CEBPB translation. These results indicated that EIF3B represents an oncogene in HNSCC. In future studies, we will provide additional information on the role of EIF3B in HNSCC perineural invasion.

## Data Availability

All relevant data can be acquired by contacting the corresponding author.
